# What Kind of Signaling Maintains Pluripotency and Viability in Human-Induced Pluripotent Stem Cells Cultured on Laminin-511 with Serum-Free Medium?

**DOI:** 10.1089/biores.2016.0001

**Published:** 2016-04-01

**Authors:** Yoshiki Nakashima, Takeshi Omasa

**Affiliations:** Department of Material and Life Science, Graduate School of Engineering, Osaka University, Osaka, Japan.

**Keywords:** cellular biology, cell culture, extracellular matrix, growth factor, stem cells

## Abstract

Xeno-free medium contains no animal-derived components, but is composed of minimal growth factors and is serum free; the medium may be supplemented with insulin, transferrin, and selenium (ITS medium). Serum-free and xeno-free culture of human-induced pluripotent stem cells (hiPSCs) uses a variety of components based on ITS medium and Dulbecco's modified Eagle's medium/Ham's nutrient mixture F12 (DMEM/F12) that contain high levels of iron salt and glucose. Culture of hiPSCs also requires scaffolding materials, such as extracellular matrix, collagen, fibronectin, laminin, proteoglycan, and vitronectin. The scaffolding component laminin-511, which is composed of α5, β1, and γ1 chains, binds to α3β1, α6β1, and α6β4 integrins on the cell membrane to induce activation of the PI3K/AKT- and Ras/MAPK-dependent signaling pathways. In hiPSCs, the interaction of laminin-511/α6β1 integrin with the cell–cell adhesion molecule E-cadherin confers protection against apoptosis through the Ras homolog gene family member A (RhoA)/Rho kinase (ROCK) signaling pathway (the major pathways for cell death) and the proto-oncogene tyrosine-protein kinase Fyn (Fyn)-RhoA-ROCK signaling pathway. The expression levels of α6β1 integrin and E-cadherin on cell membranes are controlled through the activation of insulin receptor/insulin, FGF receptor/FGF2, or activin-like kinase 5 (ALK5)-dependent TGF-β signaling. A combination of growth factors, medium constituents, cell membrane-located E-cadherin, and α6β1 integrin-induced signaling is required for pluripotent cell proliferation and for optimal cell survival on a laminin-511 scaffold. In this review, we discuss and explore the influence of growth factors on the cadherin and integrin signaling pathways in serum-free and xeno-free cultures of hiPSCs during the preparation of products for regenerative medicinal therapies. In addition, we suggest the optimum serum-free medium components for use with laminin-511, a new scaffold for hiPSC culture.

## Introduction

Initially, human pluripotent stem cell (hPSC) lines were grown in a coculture system using a layer of mitotically inactivated mouse embryonic fibroblast (MEF) feeder cells.^1–3^ However, to be clinically compliant, human-induced pluripotent stem cells (hiPSCs) need to be cultured in a medium that is serum free, chemically defined, and free of animal products (xeno free). Serum-free medium is generally composed of minimal growth factors, bovine serum albumin (BSA) and Fraction V protein; the insulin, transferrin, and selenium (ITS) medium is supplemented with ITS. Considerable efforts are being made to develop culture systems that do not require animal feeder cells and use xeno-free medium.^4–11^

hPSCs cultured in MEF-conditioned medium (MEF-CM), which is based on ITS medium, can be maintained on Matrigel,^9^ a complex mixture of matrix proteins derived from Engelbreth-Holm-Swarm mouse tumors^12–14^; this mixture includes laminin-111, collagen IV, fibronectin, and vitronectin and supports robust hPSC growth.^8,14–16^ Moreover, a combination of collagen IV, fibronectin, laminin, and vitronectin can be used to replace Matrigel for deriving and expanding hPSCs under defined culture conditions.^8^ A laminin-511 scaffold can bind α3β1, α6β1, and α6β4 integrins and is able to maintain hPSCs in the undifferentiated state in serum- and xeno-free medium under feeder cell-free culture conditions.^17–21^

In this review, we consider the effects of medium components and intracellular signal transduction in cultures using serum- and xeno-free medium and a feeder cell-free culture system. Substrates obtained from recombinant human laminin-511 offer significant improvements for establishing defined conditions for adhesion cultures of hPSCs under clinically compliant settings. We suggest that choosing the components of the culture medium and the scaffolding materials can ensure maintenance of pluripotency and cell survival through PI3K/AKT and Fyn-RhoA-ROCK signaling pathways in hiPSC culture.

## Signaling Pathways in Serum-Free Culture Systems

hiPSCs easily lose pluripotency due to a component in serum. Therefore, hiPSCs have to be cultured using serum-free culture medium. Initially, researchers examined serum albumin, a primary component of serum, and found that Dulbecco's modified Eagle's medium/Ham's nutrient mixture F12 (DMEM/F12) culture medium supplemented with serum albumin, FGF2, and the ITS medium components. ITS functioned similarly as serum-based culture media for hiPSCs. However, the serum-free medium used for the culture of hiPSCs initially contained a component derived from an animal. In addition, mouse feeder cells were also used.

### EFGF2/FGFR signaling

**FIG. 1.**
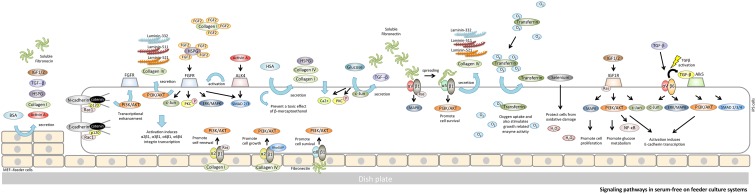
Signaling pathways in serum-free on feeder culture systems. BSA acts as a nutrient for MEFs. MEFs secrete heparan sulfate proteoglycans (HSPG), fibronectin, TGF-β, IGF1, and IGF2. HSA acts as a nutrient for hiPSCs. Albumin in the culture medium promotes expression of collagen I, collagen IV, and HSPG that detoxify β-mercaptoethanol. Collagen I binds to α2β1 integrin and transduces Ras function to activate PI3K binding to AKT; activation of the latter pathway promotes self-renewal in hiPSCs. Collagen IV binds to α2β1 integrin and transduces a RhoGAP function to activate PI3K binding to AKT; activation of the latter pathway promotes cell growth in hiPSCs. With regard to downstream functions in the IGF1R and ALK5 pathways of hiPSCs, activation induces E-cadherin transcription that recruits the PI3K/AKT and ERK/MAPK. This suggests that both PI3K/AKT and MAPK/ERK signaling are required for insulin- and TGF-β-induced E-cadherin upregulation on the cell membrane. Glucose promotes fibronectin and TGF-β manifestation through c-Jun signaling. TGF-β is activated by αVβ6 integrin. The pathways that IGF1R and ALK5 activate through unidentified growth factors, which are present in the culture medium for hiPSCs, are sufficient to maintain pluripotency in hiPSCs. Transferrin regulates cellular iron uptake and, thus, is an essential culture medium ingredient. Selenium is a component of some glutathione peroxidases; these enzymes prevent oxidative damage in cells by inducing the reduction of lipid hydroperoxides. With regard to downstream functions in the FGFR and ALK4 pathways of hiPSCs, the induction of α2β1, α3β1, α6β1, and α6β4 integrin transcription requires the FGF2-FGFR signal pathway and is reinforced by ALK4-activin A signal pathway. Collagen I and HSPG concentrate on FGF2 in the vicinity of FGFR to activate laminin-332, -511, -521, and collagen IV transcription through the PI3K/AKT signaling pathway. Fibronectin is secreted by hiPSCs and binds with α8β1 integrin to promote cell survival of hiPSCs through PI3K/AKT signaling. In addition, fibronectin can also bind to αVβ1 integrin and α5β1 integrin to promote secretion of laminin-332, -511, -521, and collagen IV from hiPSCs. The pathways activated by fibronectin-integrin signaling are responsible for the secretion of ECM, which maintains pluripotency of hiPSCs. ALK4, activin-like kinase receptor-4; BSA, bovine serum albumin; ECM, extracellular matrix; ERK, extracellular signal-regulated kinase; hiPSCs, human-induced pluripotent stem cells; FGFR, fibroblast growth factor receptor; HSA, human serum albumin; IGF, insulin-like growth factor; IGF1R, insulin-like growth factor-1 receptor; MAPK, mitogen-activated protein kinase; MEF, mouse embryonic fibroblast; PI3K, phosphatidylinositol 3-kinase; TGF-β, transforming growth factor β.

FGFR1 (fibroblast growth factor receptor 1) is the predominant FGFR in hiPSCs. Binding of FGF2 activates FGFR and major signal transduction pathways, such as all three branches of the PI3K and AKT pathways, the mitogen-activated protein kinase (MAPK) pathway, and the extracellular signal-regulated kinase (ERK)/MAPK pathway. FGF2 activates the MAP kinases ERK1/ERK2, and also PI3K/AKT and c-Jun, and stimulates signaling cascades by activation of activin A.^22,23^ Induction of α2β1, α3β1, α6β1, and α6β4 integrin transcription requires the FGF2-FGFR signal pathway and is reinforced by the ALK4-activin A signaling pathway. FGF2 signaling may act through the PI3K pathway to regulate the synthesis of pericellular laminin-332, -511, -521, and collagen IV isotypes and provide a framework for the basement membrane of hPSCs.^24^ Blocking the FGF2 pathway in hPSCs can lead to differentiation.^6,8,25^ Extracellular matrix (ECM) modulated signals may converge with FGF2 signaling and be involved in the maintenance of pluripotency (Fig. 1).

### Bovine serum albumin

BSA has many roles in cell culture; it acts as a lipid carrier, provides physical protection,^26,27^ and prevents toxic effects by β-mercaptoethanol (a reducing agent normally required for culturing cells). In addition, BSA acts as a nutrient for MEFs. Many proteins secreted by MEFs have been identified recently in mass spectrometric analyses.^28–30^ MEF secreted heparan sulfate proteoglycans (HSPG), fibronectin, transforming growth factor β (TGF-β), and insulin-like growth factors 1 (IGF1) and 2 (IGF2). In addition, it has been shown that critical products produced by MEF feeder layers include activin A^31^ (Fig. 1).

### Insulin/IGF-R signaling

The IGF-1R signaling axis is critical to cell proliferation and glucose metabolism and also induces E-cadherin transcription in hiPSCs. Upon binding insulin, IGF-1R is activated through autophosphorylation. PI3K activation then leads to an increase in phosphatidylinositol 3,4,5-trisphosphate (PIP3), which results in the activation of the critical protein AKT.^25^ This signaling axis is commonly referred to as the PI3K/AKT, nuclear factor κB (NF-κB), and jun N-terminal kinases (JNK) pathway and as c-Jun phosphorylation of IGF-1R signaling; it is ultimately responsible for the maintenance of hPSCs and hiPSCs.^32,33^ In parallel, IGF-1R signaling also promotes cell proliferation through the Ras/MAPK pathway (Fig. 1).

### Transferrin

Transferrin, a glycoprotein, is required in the culture medium to act primarily as an iron transporter and secondarily to bind endogenous iron. Transferrin enables the cells to take up oxygen and also stimulates growth-related enzyme activities. Two types of transferrin are generally added to culture media: iron-saturated transferrin (holo-transferrin) and iron-deficient transferrin (apo-transferrin) (Fig. 1).

### Selenium

Selenium, in the form of selenoproteins in particular glutathione peroxidase (Glu.Px), is an essential component of the human cellular antioxidant defense system. It also has anti-inflammatory effects by its ability to scavenge free radicals.^34^ As is evident from Figure 1, selenium is a component of some glutathione peroxidases; these enzymes prevent oxidative damage in cells by inducing the reduction of lipid hydroperoxides.

### Glucose

In hPSCs, the c-jun N-terminal kinase (JNK) inhibitor SP600125 blocks fibronectin expression induced by high glucose and inhibits cell cycle regulatory protein expression induced by high glucose-mediated TGF-β expression.^35^ High glucose levels (4.5 g/L) also increase intracellular calcium (Ca^2+^) concentration, PKC phosphorylation, and TGF-β expression in hPSCs. Thus, c-Jun is necessary for expression of fibronectin and TGF-β (Fig. 1).

## Signaling Pathways in Feeder Cell-Free Culture Systems

When hiPSCs are cultured on an MEF feeder layer, the MEFs provide proteins, such as activin A, TGF-β, IGF1 and 2 (IGF2), and glycans, including HSPG, fibronectin, collagen I, collagen IV, and laminin. These proteins and glycans are necessary for hiPSC attachment, proliferation, and self-renewal^36^ (Fig. 1). Matrigel, which includes laminin-111, collagen IV, fibronectin, and vitronectin, can be used to replace the MEF feeder layer and support robust hPSC growth.^12–14^

### Activin A/ALK4 signaling

**FIG. 2.**
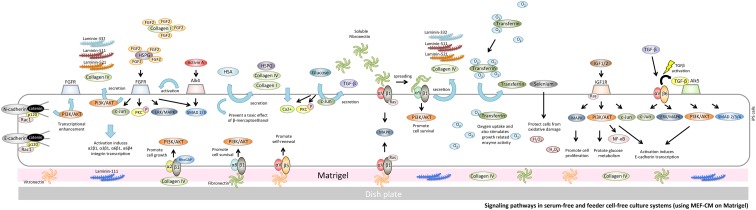
Signaling pathways in serum-free and feeder cell-free culture systems (using MEF-CM on Matrigel). Matrigel includes laminin-111, collagen IV, fibronectin, and vitronectin and supports robust hPSC growth. Collagen IV binds to α2β1 integrin and transduces a RhoGAP function to activate PI3K binding to AKT; activation of the latter pathway promotes cell growth in hiPSCs. Vitronectin supports the maintenance of hPSCs through αVβ5 integrin. αVβ1 integrin is able to recognize vitronectin and fibrinogen. Fibronectin can also bind to αVβ1 integrin and α5β1 integrin to promote secretion of laminin-332, -511, -521, and collagen IV from hiPSCs. hPSC, human pluripotent stem cells; MEF-CM, MEF-conditioned medium.

Activin A has a role in a wide range of cellular processes from cell proliferation and differentiation to apoptosis. Initially, activin A binds to type II activin A receptors (ActRIIA or ActRIIB) and then recruits type IB activin A receptor (ALK4). ALK4 interacts with and phosphorylates the SMAD family members 2 and 3 (SMAD2 and SMAD3). Vallier et al.^37–39^ demonstrated that the cooperation of the activin A pathway and FGF2 is necessary for the maintenance of pluripotency in hPSCs.^40^ When active, PI3K/AKT establishes conditions where activin A/SMAD2/3 stimulate self-renewal in hiPSCs by activating target genes, including Nanog.^41,42^ This stimulatory effect involves the interaction of activin A and SMAD2/3, and may also require upregulation of FGF2 pathways^39,43^ (Fig. 2).

### TGF-β/ALK5 signaling

TGF-β type 1 receptor, also known as ALK5, phosphorylates SMAD2/3 and forms a complex with SMAD4 that translocates to the nucleus. The complex induces transcription of TGF-β-dependent genes that regulate signaling pathways involved in pluripotency and self-renewal, such as the MAPK and PI3K/AKT pathways. Signaling through these pathways supports proliferation and continued pluripotency in hiPSCs^44^ (Fig. 2).

## Signaling Pathways in Xeno-Free Culture Systems

Animal serum contains xenogeneic antigens, and cells cultured in serum have the potential to induce immune reactions in recipients and also raise the risk of transmission of animal viruses, prions, and zoonotic infections. Thus, animal serum is not desirable for clinical cell production or for transplantation therapies. Laminin-511 scaffold can bind α3β1, α6β1, and α6β4 integrins and can be used to maintain hPSCs in the undifferentiated state under serum and xeno-free conditions in a feeder cell-free culture system.^17–21^

### Recombinant human albumin

**FIG. 3.**
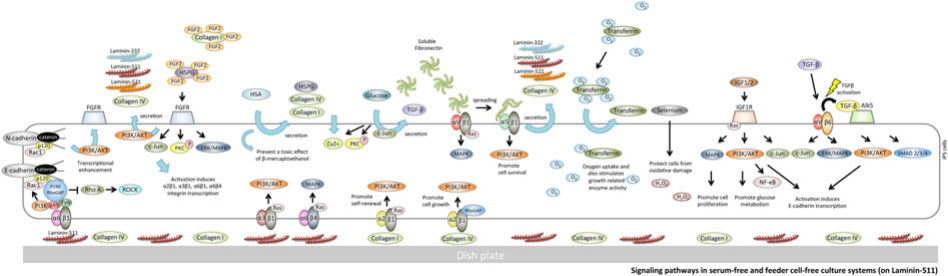
Signaling pathways in serum-free and feeder cell-free culture systems (on laminin-511). Cadherin is activated by a cell junction. When N-cadherin is activated, transcriptional enhancement of FGFR occurs. As a result, the FGF2 signal pathway is activated. This signaling pathway has an important role in the expression of FGFR attachment to the cell membrane. In cultures using a laminin-511 scaffold, the E-cadherin-α6β1 integrin signal pathway is required to control cell death in hiPSCs. PI3K signaling promotes expression of Rac1. Rac1 binds to cadherins, and cadherins prevent endocytosis. When α6β1 integrin is combined with laminin-511, Fyn-RhoA-ROCK signaling is induced. As a result, hiPSC death caused by ROCK is repressed. Laminin-511 can directly bind to α3β1 integrin and, thereby, activate the PI3K/AKT signaling pathway through Ras. In addition, linkage of α6β4 activates the MAP kinase pathways directly through Ras.

Human albumin has many biological and physical roles in hiPSC culture and is necessary for StemFit medium (Ajinomoto Co., Inc.) to enable the efficient formation of colonies by hiPSCs on recombinant laminin-511.^45^ However, albumin in the culture medium promotes the secretion of ECM molecules such as collagen I, collagen IV, and HSPG. Consequently, protein lytic enzymes such as the trypsin are necessary to break the strong cell-to-plate adhesion (Fig. 3).

### Recombinant vitronectin scaffold

Recombinant vitronectin as a glycoprotein can function as a chemically defined matrix component in hiPSCs. Vitronectin is a functionally defined substrate that supports hPSCs self-renewal through αVβ5 integrin in albumin-free medium such as Essential 8 medium (Thermo Fisher Scientific). It is possible to dislodge cells from the culture plate using only EDTA; thus, integrins on the cell surface are not damaged by trypsin treatment.

### Recombinant laminin-511 scaffold

The scaffolding component laminin-511, which is composed of α5, β1, and γ1 chains, binds to α3β1, α6β1, and α6β4 integrins on the cell membrane to induce activation of the PI3K/AKT- and Ras/MAPK-dependent signaling pathways. In hiPSCs, the interaction of laminin-511/α6β1 integrin with the cell–cell adhesion molecule E-cadherin confers protection against apoptosis through the Ras homolog gene family member A (RhoA)/Rho kinase (ROCK) signaling pathway (the major pathway for cell death) and the proto-oncogene tyrosine-protein kinase Fyn (Fyn)-RhoA-ROCK signaling pathway.

## Signaling Pathways in Cultures Using Laminin-511

### Cadherin

Cell adhesion molecules (CAMs), such as cadherins and integrins, stimulate intercell adhesion. The “classical” CAMs include epithelial (E-), neural (N-), and placental (P-) cadherins and, as their names suggest, they tend to be expressed in a tissue-specific pattern.^46–52^ In addition to their role in cell–cell adhesion, cadherins may also function in signaling pathways between the cell membrane and the nucleus.^53^

#### E-cadherin signaling

E-cadherin, a Ca^2+^-dependent cell–cell adhesion molecule,^50,54^ is stabilized at the cell surface by its link to the actin cytoskeleton through β-catenin and α-catenin and is essential for PSC intercellular adhesion and colony formation.^55,56^ It has been shown that functional interaction between Rac1, a small Rho family GTPase, and E-cadherin is responsible for regulating hPSC self-renewal.^57,58^ E-cadherin-mediated adhesion is commonly associated with β-catenin signaling and also stimulates PI3K/AKT signaling.^59,60^ The ability of the PI3K/AKT pathway to promote cell survival indicates a broader role for Rho family GTPases through p120-catenin.^61,62^ The PI3K inhibitor wortmannin prevents E-cadherin-dependent Rac1 activation, but does not interfere with the intracellular distribution of Rac1 or E-cadherin.^63^ The activation of Rac1 through PI3K by E-cadherin-mediated cell–cell adhesion involves at least two steps as follows: (1) recruitment of Rac1 to E-cadherin mediates cell–cell contact sites and (2) Rac1 activation by a guanine nucleotide exchange factor.^64^ E-cadherin levels at adherens junctions are regulated by endocytosis and recycling; activated Rac1 reduces E-cadherin endocytosis and, thus, increases the levels of cell surface E-cadherin and, consequently, promotes cell–cell adhesion. Ectopic overexpression of E-cadherin also increases the rate of survival in dissociated hPSCs.^65^ However, when dissociated hPSCs are grown on E-cadherin-coated plates, they form membrane protrusions and have a lower rate of survival. Thus, E-cadherin interactions alone are not the only factor in cell survival.^66^ Transcription factors for E-cadherin expression appear to act downstream in various signaling pathways, such as in the TGF-β, FGF2, NF-κB, and integrin cascades^67^ (Fig. 3).

#### N-cadherin signaling

N-cadherin induces FGFR upregulation^68^ and has also been shown to stimulate FGF2 signaling.^69^ N-cadherin adhesion can lead to PI3K-mediated activation of AKT,^70,71^ and activated AKT signaling can stimulate β-catenin signaling. The E-cadherin function can be replaced by N-cadherin in the rescue of the undifferentiated pluripotent iPSC state^72^ (Fig. 3).

#### P-cadherin signaling

P-cadherin controls cell-to-laminin adhesion by modulating the expression and activation of the laminin receptor α6β4 integrin heterodimer.^73^ Importantly, it has been reported that α6β4 integrin (adhering to laminin-332-containing α3, β3, and γ2 chains) promotes survival and invasion by activating the PI3K/AKT pathway.^74–76^ α6 Integrin activation induces P-cadherin transcription.^77^ P-cadherin results in the translocation of p120ctn from the plasma membrane to the cytoplasm, thereby activating Rac1.^78^

### Integrin

Integrins are transmembrane proteins that have a wide range of functions, including adhesion and cell–cell interactions.^79^ They are heterodimeric molecules consisting of α and β chains. The 24 different heterodimeric combinations of integrin subunits in vertebrates have different ligand binding preferences. Based on their ligand specificities, the integrin family can be divided into three subgroups with respect to hPSC culture: collagen receptors (α2β1 and α11β1), laminin receptors (α3β1, α6β1, α7β1, and α6β4), and RGD (Arg-Gly-Asp) receptors (α5β1, α8β1, αVβ1, αVβ5, αVβ6, and αIIbβ3).^36,80–82^

### Collagen receptors (α2β1 and α11β1)

Total collagen complex, including types I, II, III, and IV, can induce an increased proliferative capacity in iPSCs.^83^ The effect of collagen I and IV on cell migration is mediated by α2β1 integrin.^84,85^ Collagen is not included in cultures using a laminin-511 scaffold, although hiPSC lines do express both collagen I and IV.^86,87^ Albumin in the culture medium promotes collagen secretion by hiPSCs. Collagen I has been reported to stabilize FGF2, similar to HSPG, for floating cultures of hiPSCs.^88^

#### α2β1 integrin/collagen I/IV signaling

Collagen I-bound α2β1 integrin stimulates the self-renewal of PSCs by increasing the PI3K/AKT signal through the GTP Ras pathway.^89^ Collagen IV pretreatment enhances *in vivo* growth of undifferentiated PSCs without affecting their pluripotency through α2β1 integrin-mediated actin remodeling and the regulatory molecules PI3K and Rho-family GTPases^84^ (Fig. 3).

### Laminin receptors (α3β1, α6β1, α7β1, and α6β4)

Laminins are large glycoprotein molecules consisting of α, β, and γ chains. To date, five α, three β, and three γ chains have been identified. Laminin expression is present in early embryos and in pluripotent mouse and human ESCs. Cultured pluripotent hPSCs express a laminin-511 heterotrimer (α5β1γ1), consisting of α5, β1, and γ1 laminin chains.^90^

#### α3β1 integrin/laminin-332, -511, -521 signaling

The role of integrin α3β1 in cell migration is unclear. Laminin-332 drives PI3K activation and cell survival.^76^ Gu et al.^91^ demonstrated that laminin-511 and laminin-521 directly bind to α3β1 integrin and, thereby, activate the PI3K/AKT signaling pathway (Fig. 3). However, it is not yet clear whether laminin-511 and laminin-332 use the same or different binding sites in these receptors.

#### α6β1 integrin/laminin-332, -511, -521 signaling

Laminin-511 and laminin-521 maintain hPSC pluripotency after α6β1 integrin signaling to induce the PI3K/AKT signaling pathway.^90,92^ In cultures using a laminin-511 scaffold, the interaction between laminin-511 and α6β1 integrin partly compensates for the ROCK inhibitor, Y-27632. The proto-oncogene tyrosine-protein kinase Fyn^93^ associates with the p85 subunit of PI3K. The Fyn-RhoA-ROCK signaling cascade^94^ is of interest since Fyn directly associates with α6β1-integrin.^95,96^ PSCs interact with laminin-511 through β1-integrins, mostly α6β1. Strong Fyn-RhoA-ROCK signaling is expected in cultures using laminin-511 as the scaffold (Fig. 3).

#### α6β4 integrin/laminin-332, -511, -521 signaling

Upon binding to laminin-332, the integrin α6β4 can organize hemidesmosomes, which are essential for stable cell adhesion.^97,98^ Furthermore, FGF2 upregulates the expression of α2β1, α3β1, α6β1, and α6β4 integrins in cells cultured *in vitro*.^99^ Therefore, it is believed that α6β4 integrin in hiPSC membranes increases if concentrated FGF2 is added to the culture medium (Fig. 3).

### RGD receptors (α5β1, α8β1, αVβ1, αVβ5, and αVβ6)

The tripeptide sequence arginine-glycine-aspartic acid (RGD) has been suggested to be a recognition sequence for integrins.^100^ This peptide sequence has been identified in many ECM proteins such as fibronectin, vitronectin, fibrinogen, von Willebrand factor, thrombospondin, laminin, entactin, tenascin, osteopontin, and bone sialoprotein.^101,102^

#### α5β1 integrin/fibronectin signaling

Human fibronectin, which binds to integrin α5β1,^103^ supports hPSC expansion in serum-free medium.^104^ α5β1 integrin binds soluble fibronectin efficiently; first, the α5 integrin binds to the synergy site to cause a conformational change in the fibronectin; second, β1 integrin binds to the exposed RGD sequence.^105^ Overexpression of α5β1 provides considerable protection against several proapoptotic stimuli. Furthermore, the protective effects of α5β1 are mediated through a PI3K/AKT-dependent pathway^106^ (Fig. 3).

#### α8β1 integrin/fibronectin signaling

α8β1 integrin, a cell adhesion receptor that links the cytoskeleton to ECM proteins, not only mediates mechanical and chemical signals from the matrix but also regulates cell adhesion through bidirectional membrane transduction.^107^ Under serum deprivation conditions, α8β1 can interact with fibronectin to increase cell survival rates so that α8-mediated survival is mediated by the PI3K pathway^108^ (Fig. 2).

#### αVβ1 integrin/fibronectin signaling

αVβ1 has been described as a receptor for fibronectin,^109^ vitronectin,^110,111^ or collagen.^112^ αVβ1 integrin is able to recognize vitronectin and fibrinogen and also cooperate with α5β1 to mediate attachment to and spreading on fibronectin;^91,113^ Ras/MEK/MAPK signaling and kinases are also stimulated by integrin ligation (Fig. 2).

#### αVβ5 integrin/vitronectin signaling

**FIG. 4.**
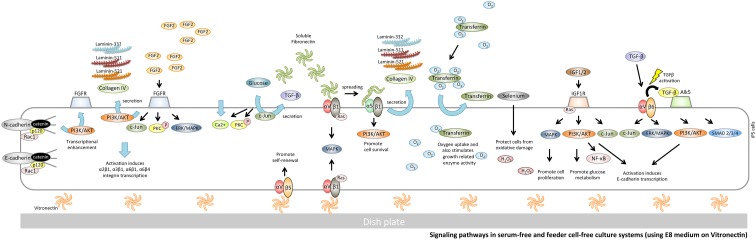
Signaling pathways in serum-free and feeder cell-free culture systems (using E8 medium on vitronectin). Recombinant vitronectin is a functionally defined substrate that supports hPSCs self-renewal through αVβ5 integrin. αVβ1 has been described as a receptor for vitronectin through Ras/MEK/MAPK signaling, and kinases are also stimulated by integrin ligation. This culture system does not need albumin; as collagen I and HSPG do not concentrate on FGF2, it is necessary to have a high concentration of FGF2 in the culture medium.

Recombinant vitronectin is a functionally defined substrate that supports hPSCs self-renewal through αVβ5 integrin^36^ (Fig. 4). αVβ5 activates latent TGF-β.^114^ Chen et al. enhanced the attachment and survival of hPSCs on vitronectin-coated surfaces by excising the N-terminal and/or C-terminal fragments of full-length vitronectin.^32^ It has also been reported that vitronectin and one of its variants (VTN-NC) support the maintenance of hPSCs through αVβ5 integrin.^32,36,115^

#### αVβ6 integrin/TGF-β signaling

αVβ6 integrin has a key role in regulating TGF-β activation of ALK5, a TGF-β type I receptor.^114,116,117^ The association with αVβ6 integrin expression may be linked to TGF-β activation, which has an important role in intracellular signaling. TGF-β activates c-Jun and Erk through αVβ6 integrin. TGF-β is an essential additive in culture medium using a laminin-511 scaffold (Fig. 3).

## Conclusion

This review highlights the components that are suitable for serum- and xeno-free media and feeder cell-free culture systems that use laminin-511 as the scaffold. The binding of α6β1 with laminin-511 and the interaction with E-cadherin stimulate Fyn-RhoA-ROCK signaling to act as a deactivation ROCK signal. These results indicate that the signaling pathways induced by a laminin-511 scaffold depend on PI3K signaling through E-cadherin-mediated cell–cell adhesion and that the Fyn-RhoA-ROCK signaling cascade is associated with α6β1 integrin-laminin-511 adhesion.

## Abbreviations Used

ALK: activin-like kinase
BSA: bovine serum albumin
CAMs: cell adhesion molecules
ECM: extracellular matrix
ERK: extracellular signal-regulated kinase
FGFR: fibroblast growth factor receptor
HSPG: heparan sulfate proteoglycans
hiPSCs: human-induced pluripotent stem cells
hPSC: human pluripotent stem cell
IGF: insulin-like growth factor
ITS: insulin, transferrin, and selenium
MAPK: mitogen-activated protein kinase
MEF: mouse embryonic fibroblast
NF-κB: nuclear factor κB
TGF-β: transforming growth factor β

## References

[1] Reubinoff BE, Pera MF, Fong CY, et al. Embryonic stem cell lines from human blastocysts: somatic differentiation in vitro. Nat Biotechnol. 2000;18:399–404 10748519 10.1038/74447

[2] Takahashi K, Tanabe K, Ohnuki M, et al. Induction of pluripotent stem cells from adult human fibroblasts by defined factors. Cell. 2007;131:861–872 18035408 10.1016/j.cell.2007.11.019

[3] Thomson JA, Itskovitz-Eldor J, Shapiro SS, et al. Embryonic stem cell lines derived from human blastocysts. Science. 1998;282:1145–1147 9804556 10.1126/science.282.5391.1145

[4] Beattie GM, Lopez AD, Bucay N, et al. Activin A maintains pluripotency of human embryonic stem cells in the absence of feeder layers. Stem Cells. 2005;23:489–495 15790770 10.1634/stemcells.2004-0279

[5] Klimanskaya I, Chung Y, Meisner L, et al. Human embryonic stem cells derived without feeder cells. Lancet. 2005;365:1636–1641 15885296 10.1016/S0140-6736(05)66473-2

[6] Levenstein ME, Ludwig TE, Xu RH, et al. Basic fibroblast growth factor support of human embryonic stem cell self-renewal. Stem Cells. 2006;24:568–574 16282444 10.1634/stemcells.2005-0247 PMC4615709

[7] Li Y, Powell S, Brunette E, et al. Expansion of human embryonic stem cells in defined serum-free medium devoid of animal-derived products. Biotechnol Bioeng. 2005;91:688–698 15971228 10.1002/bit.20536

[8] Ludwig TE, Levenstein ME, Jones JM, et al. Derivation of human embryonic stem cells in defined conditions. Nat Biotechnol. 2006;24:185–187 16388305 10.1038/nbt1177

[9] Xu C, Inokuma MS, Denham J, et al. Feeder-free growth of undifferentiated human embryonic stem cells. Nat Biotechnol. 2001;19:971–974 11581665 10.1038/nbt1001-971

[10] Xu C, Rosler E, Jiang J, et al. Basic fibroblast growth factor supports undifferentiated human embryonic stem cell growth without conditioned medium. Stem Cells. 2005;23:315–323 15749926 10.1634/stemcells.2004-0211

[11] Xu RH, Peck RM, Li DS, et al. Basic FGF and suppression of BMP signaling sustain undifferentiated proliferation of human ES cells. Nat Meth. 2005;2:185–190 10.1038/nmeth744 15782187

[12] Stover AE, Schwartz PH Adaptation of human pluripotent stem cells to feeder-free conditions in chemically defined medium with enzymatic single-cell passaging. Methods Mol Biol. 2011;767:137–146 21822872 10.1007/978-1-61779-201-4_10 PMC3674824

[13] Totonchi M, Taei A, Seifinejad A, et al. Feeder- and serum-free establishment and expansion of human induced pluripotent stem cells. Int J Dev Biol. 2010;54:877–886 19876814 10.1387/ijdb.092903mt

[14] Kleinman HK, McGarvey ML, Liotta LA, et al. Isolation and characterization of type IV procollagen, laminin, and heparan sulfate proteoglycan from the EHS sarcoma. Biochemistry. 1982;21:6188–6193 6217835 10.1021/bi00267a025

[15] Bissell DM, Arenson DM, Maher JJ, et al. Support of cultured hepatocytes by a laminin-rich gel. Evidence for a functionally significant subendothelial matrix in normal rat liver. J Clin Invest. 1987;79:801–812 3546380 10.1172/JCI112887 PMC424203

[16] Horejs CM, Serio A, Purvis A, et al. Biologically-active laminin-111 fragment that modulates the epithelial-to-mesenchymal transition in embryonic stem cells. Proc Natl Acad Sci USA. 2014;111:5908–5913 24706882 10.1073/pnas.1403139111 PMC4000797

[17] Gao J, DeRouen MC, Chen CH, et al. Laminin-511 is an epithelial message promoting dermal papilla development and function during early hair morphogenesis. Gene Dev. 2008;22:2111–2124 18676816 10.1101/gad.1689908 PMC2492752

[18] Hakala H, Rajala K, Ojala M, et al. Comparison of biomaterials and extracellular matrices as a culture platform for multiple, independently derived human embryonic stem cell lines. Tissue Eng Part A. 2009;15:1775–1785 19132919 10.1089/ten.tea.2008.0316

[19] International Stem Cell Initiative Consortium, Akopian V, Andrews PW, et al. Comparison of defined culture systems for feeder cell free propagation of human embryonic stem cells. In Vitro Cell Dev Biol Anim. 2010;46:247–258 20186512 10.1007/s11626-010-9297-z PMC2855804

[20] Chin AC, Padmanabhan J, Oh SK, et al. Defined and serum-free media support undifferentiated human embryonic stem cell growth. Stem Cells Dev. 2010;19:753–761 19686051 10.1089/scd.2009.0210

[21] Rajala K, Hakala H, Panula S, et al. Testing of nine different xeno-free culture media for human embryonic stem cell cultures. Hum Reprod. 2007;22:1231–1238 17251355 10.1093/humrep/del523

[22] Feng GS Shp2-mediated molecular signaling in control of embryonic stem cell self-renewal and differentiation. Cell Res. 2007;17:37–41 17211446 10.1038/sj.cr.7310140

[23] Saxena S, Hanwate M, Deb K, et al. FGF2 secreting human fibroblast feeder cells: a novel culture system for human embryonic stem cells. Mol Reprod Dev. 2008;75:1523–1532 18318041 10.1002/mrd.20895

[24] Li XF, Chen YL, Scheele S, et al. Fibroblast growth factor signaling and basement membrane assembly are connected during epithelial morphogenesis of the embryoid body. J Cell Biol. 2001;153:811–822 11352941 10.1083/jcb.153.4.811 PMC2192393

[25] Bendall SC, Stewart MH, Menendez P, et al. IGF and FGF cooperatively establish the regulatory stem cell niche of pluripotent human cells in vitro. Nature. 2007;448:1015–1021 17625568 10.1038/nature06027

[26] Garcia-Gonzalo FR, Izpisua Belmonte JC Albumin-associated lipids regulate human embryonic stem cell self-renewal. PLoS One. 2008;3:e1384 18167543 10.1371/journal.pone.0001384 PMC2148252

[27] Rothschild MA, Oratz M, Schreiber SS Regulation of albumin metabolism. Annu Rev Med. 1975;26:91–104 1096784 10.1146/annurev.me.26.020175.000515

[28] Bendall SC, Hughes C, Campbell JL, et al. An enhanced mass spectrometry approach reveals human embryonic stem cell growth factors in culture. Mol Cell Proteomics. 2009;8:421–432 18936058 10.1074/mcp.M800190-MCP200 PMC2649806

[29] Sarkar P, Collier TS, Randall SM, et al. The subcellular proteome of undifferentiated human embryonic stem cells. Proteomics. 2012;12:421–430 22144211 10.1002/pmic.201100507

[30] Sarkar P, Randall SM, Muddiman DC, et al. Targeted proteomics of the secretory pathway reveals the secretome of mouse embryonic fibroblasts and human embryonic stem cells. Mol Cell Proteomics. 2012;11:1829–1839 22984290 10.1074/mcp.M112.020503 PMC3518120

[31] Xiao L, Yuan X, Sharkis SJ Activin A maintains self-renewal and regulates fibroblast growth factor, Wnt, and bone morphogenic protein pathways in human embryonic stem cells. Stem Cells. 2006;24:1476–1486 16456129 10.1634/stemcells.2005-0299

[32] Chen G, Gulbranson DR, Hou Z, et al. Chemically defined conditions for human iPSC derivation and culture. Nature methods. 2011;8:424–429 21478862 10.1038/nmeth.1593 PMC3084903

[33] Wang L, Schulz TC, Sherrer ES, et al. Self-renewal of human embryonic stem cells requires insulin-like growth factor-1 receptor and ERBB2 receptor signaling. Blood. 2007;110:4111–4119 17761519 10.1182/blood-2007-03-082586 PMC2190616

[34] Duntas LH Selenium and inflammation: underlying anti-inflammatory mechanisms. Horm Metab Res. 2009;41:443–447 19418416 10.1055/s-0029-1220724

[35] Kim YH, Ryu JM, Lee YJ, et al. Fibronectin synthesis by high glucose level mediated proliferation of mouse embryonic stem cells: involvement of ANG II and TGF-beta1. J Cell Physiol. 2010;223:397–407 20112290 10.1002/jcp.22048

[36] Braam SR, Zeinstra L, Litjens S, et al. Recombinant vitronectin is a functionally defined substrate that supports human embryonic stem cell self-renewal via alphavbeta5 integrin. Stem Cells. 2008;26:2257–2265 18599809 10.1634/stemcells.2008-0291

[37] Vallier L, Reynolds D, Pedersen RA Nodal inhibits differentiation of human embryonic stem cells along the neuroectodermal default pathway. Dev Biol. 2004;275:403–421 15501227 10.1016/j.ydbio.2004.08.031

[38] Vallier L, Mendjan S, Brown S, et al. Activin/Nodal signalling maintains pluripotency by controlling Nanog expression. Development. 2009;136:1339–1349 19279133 10.1242/dev.033951 PMC2687465

[39] Vallier L, Alexander M, Pedersen RA Activin/Nodal and FGF pathways cooperate to maintain pluripotency of human embryonic stem cells. J Cell Sci. 2005;118:4495–4509 16179608 10.1242/jcs.02553

[40] Kinehara M, Kawamura S, Tateyama D, et al. Protein kinase C regulates human pluripotent stem cell self-renewal. PLoS One. 2013;8:e54122 23349801 10.1371/journal.pone.0054122 PMC3549959

[41] Ogawa K, Saito A, Matsui H, et al. Activin-Nodal signaling is involved in propagation of mouse embryonic stem cells. J Cell Sci. 2007;120:55–65 17182901 10.1242/jcs.03296

[42] Singh AM, Reynolds D, Cliff T, et al. Signaling network crosstalk in human pluripotent cells: a Smad2/3-regulated switch that controls the balance between self-renewal and differentiation. Cell Stem Cell. 2012;10:312–326 22385658 10.1016/j.stem.2012.01.014 PMC3294294

[43] Babaie Y, Herwig R, Greber B, et al. Analysis of Oct4-dependent transcriptional networks regulating self-renewal and pluripotency in human embryonic stem cells. Stem Cells. 2007;25:500–510 17068183 10.1634/stemcells.2006-0426

[44] Yamasaki S, Taguchi Y, Shimamoto A, et al. Generation of human induced pluripotent stem (Ips) cells in serum- and feeder-free defined culture and TGF-Beta1 regulation of pluripotency. PLoS One. 2014;9:e87151 24489856 10.1371/journal.pone.0087151 PMC3906124

[45] Nakagawa M, Taniguchi Y, Senda S, et al. A novel efficient feeder-free culture system for the derivation of human induced pluripotent stem cells. Sci Rep. 2014;4:3594 24399248 10.1038/srep03594 PMC3884228

[46] Gumbiner BM Cell adhesion: the molecular basis of tissue architecture and morphogenesis. Cell. 1996;84:345–357 8608588 10.1016/s0092-8674(00)81279-9

[47] Takeichi M Cadherin cell adhesion receptors as a morphogenetic regulator. Science. 1991;251:1451–1455 2006419 10.1126/science.2006419

[48] Geiger B, Ayalon O Cadherins. Annu Rev Cell Biol. 1992;8:307–332 1476802 10.1146/annurev.cb.08.110192.001515

[49] Kemler R From cadherins to catenins: cytoplasmic protein interactions and regulation of cell adhesion. Trends Genet. 1993;9:317–321 8236461 10.1016/0168-9525(93)90250-l

[50] Takeichi M Morphogenetic roles of classic cadherins. Curr Opin Cell Biol. 1995;7:619–627 8573335 10.1016/0955-0674(95)80102-2

[51] Hatta K, Takeichi M Expression of N-cadherin adhesion molecules associated with early morphogenetic events in chick development. Nature. 1986;320:447–449 3515198 10.1038/320447a0

[52] Nose A, Takeichi M A novel cadherin cell adhesion molecule: its expression patterns associated with implantation and organogenesis of mouse embryos. J Cell Biol. 1986;103:2649–2658 3539943 10.1083/jcb.103.6.2649 PMC2114609

[53] Cavallaro U, Christofori G Cell adhesion and signalling by cadherins and Ig-CAMs in cancer. Nat Rev Cancer. 2004;4:118–132 14964308 10.1038/nrc1276

[54] Gumbiner BM Regulation of cadherin-mediated adhesion in morphogenesis. Nat Rev Mol Cell Bio. 2005;6:622–634 16025097 10.1038/nrm1699

[55] Larue L, Antos C, Butz S, et al. A role for cadherins in tissue formation. Development. 1996;122:3185–3194 8898231 10.1242/dev.122.10.3185

[56] Dang SM, Gerecht-Nir S, Chen J, et al. Controlled, scalable embryonic stem cell differentiation culture. Stem Cells. 2004;22:275–282 15153605 10.1634/stemcells.22-3-275

[57] Li L, Wang SA, Jezierski A, et al. A unique interplay between Rap1 and E-cadherin in the endocytic pathway regulates self-renewal of human embryonic stem cells. Stem Cells. 2010;28:247–257 20039365 10.1002/stem.289

[58] Ohgushi M, Sasai Y Lonely death dance of human pluripotent stem cells: ROCKing between metastable cell states. Trends Cell Biol. 2011;21:274–282 21444207 10.1016/j.tcb.2011.02.004

[59] De Santis G, Miotti S, Mazzi M, et al. E-cadherin directly contributes to PI3K/AKT activation by engaging the PI3K-p85 regulatory subunit to adherens junctions of ovarian carcinoma cells. Oncogene. 2009;28:1206–1217 19151754 10.1038/onc.2008.470

[60] Pece S, Chiariello M, Murga C, et al. Activation of the protein kinase Akt/PKB by the formation of E-cadherin-mediated cell-cell junctions. Evidence for the association of phosphatidylinositol 3-kinase with the E-cadherin adhesion complex. J Biol Chem. 1999;274:19347–19351 10383446 10.1074/jbc.274.27.19347

[61] Goodwin M, Kovacs EM, Thoreson MA, et al. Minimal mutation of the cytoplasmic tail inhibits the ability of E-cadherin to activate Rac but not phosphatidylinositol 3-kinase: direct evidence of a role for cadherin-activated Rac signaling in adhesion and contact formation. J Biol Chem. 2003;278:20533–20539 12672818 10.1074/jbc.M213171200

[62] Thiery JP Cell adhesion in development: a complex signaling network. Curr Opin Genet Dev. 2003;13:365–371 12888009 10.1016/s0959-437x(03)00088-1

[63] Nakagawa M, Fukata M, Yamaga M, et al. Recruitment and activation of Rac1 by the formation of E-cadherin-mediated cell-cell adhesion sites. J Cell Sci. 2001;114:1829–1838 11329369 10.1242/jcs.114.10.1829

[64] Fukata M, Kaibuchi K Rho-family GTPases in cadherin-mediated cell-cell adhesion. Nat Rev Mol Cell Biol. 2001;2:887–897 11733768 10.1038/35103068

[65] Rizzino A Stimulating progress in regenerative medicine: improving the cloning and recovery of cryopreserved human pluripotent stem cells with ROCK inhibitors. Regen Med. 2010;5:799–807 20868334 10.2217/rme.10.45 PMC3037821

[66] Ohgushi M, Matsumura M, Eiraku M, et al. Molecular pathway and cell state responsible for dissociation-induced apoptosis in human pluripotent stem cells. Cell Stem Cell. 2010;7:225–239 20682448 10.1016/j.stem.2010.06.018

[67] Soncin F, Ward CM The function of e-cadherin in stem cell pluripotency and self-renewal. Genes (Basel). 2011;2:229–259 24710147 10.3390/genes2010229 PMC3924836

[68] Qian X, Anzovino A, Kim S, et al. N-cadherin/FGFR promotes metastasis through epithelial-to-mesenchymal transition and stem/progenitor cell-like properties. Oncogene. 2014;33:3411–3421 23975425 10.1038/onc.2013.310 PMC4051865

[69] Stenzel D, Nye E, Nisancioglu M, et al. Peripheral mural cell recruitment requires cell-autonomous heparan sulfate. Blood. 2009;114:915–924 19398718 10.1182/blood-2008-10-186239

[70] Tran NL, Adams DG, Vaillancourt RR, et al. Signal transduction from N-cadherin increases Bcl-2. Regulation of the phosphatidylinositol 3-kinase/Akt pathway by homophilic adhesion and actin cytoskeletal organization. J Biol Chem. 2002;277:32905–32914 12095980 10.1074/jbc.M200300200

[71] Zhang JN, Shemezis JR, McQuinn ER, et al. AKT activation by N-cadherin regulates beta-catenin signaling and neuronal differentiation during cortical development. Neural Dev. 2013;8 23618343 10.1186/1749-8104-8-7 PMC3658902

[72] Bedzhov I, Alotaibi H, Basilicata MF, et al. Adhesion, but not a specific cadherin code, is indispensable for ES cell and induced pluripotency. Stem Cell Res. 2013;11:1250–1263 24036274 10.1016/j.scr.2013.08.009

[73] Vieira AF, Ribeiro AS, Dionisio MR, et al. P-cadherin signals through the laminin receptor alpha6beta4 integrin to induce stem cell and invasive properties in basal-like breast cancer cells. Oncotarget. 2014;5:679–692 24553076 10.18632/oncotarget.1459 PMC3996674

[74] Shaw LM, Rabinovitz I, Wang HH, et al. Activation of phosphoinositide 3-OH kinase by the alpha6beta4 integrin promotes carcinoma invasion. Cell. 1997;91:949–960 9428518 10.1016/s0092-8674(00)80486-9

[75] Bachelder RE, Ribick MJ, Marchetti A, et al. p53 inhibits alpha 6 beta 4 integrin survival signaling by promoting the caspase 3-dependent cleavage of AKT/PKB. J Cell Biol. 1999;147:1063–1072 10579725 10.1083/jcb.147.5.1063 PMC2169339

[76] Marinkovich MP Tumour microenvironment: laminin 332 in squamous-cell carcinoma. Nat Rev Cancer. 2007;7:370–380 17457303 10.1038/nrc2089

[77] Deugnier MA, Faraldo MM, Rousselle P, et al. Cell-extracellular matrix interactions and EGF are important regulators of the basal mammary epithelial cell phenotype. J Cell Sci. 1999;112:1035–1044 10198285 10.1242/jcs.112.7.1035

[78] Cheung LW, Leung PC, Wong AS Cadherin switching and activation of p120 catenin signaling are mediators of gonadotropin-releasing hormone to promote tumor cell migration and invasion in ovarian cancer. Oncogene. 2010;29:2427–2440 20118984 10.1038/onc.2009.523

[79] Giancotti FG, Ruoslahti E Integrin signaling. Science. 1999;285:1028–1032 10446041 10.1126/science.285.5430.1028

[80] Brafman DA, Phung C, Kumar N, et al. Regulation of endodermal differentiation of human embryonic stem cells through integrin-ECM interactions. Cell Death Differ. 2013;20:369–381 23154389 10.1038/cdd.2012.138 PMC3569984

[81] Evseenko D, Schenke-Layland K, Dravid G, et al. Identification of the critical extracellular matrix proteins that promote human embryonic stem cell assembly. Stem Cells Dev. 2009;18:919–928 19021502 10.1089/scd.2008.0293

[82] Hynes RO Integrins: bidirectional, allosteric signaling machines. Cell. 2002;110:673–687 12297042 10.1016/s0092-8674(02)00971-6

[83] Chang BS, Choi YJ, Kim JH Collagen complexes increase the efficiency of iPS cells generated using fibroblasts from adult mice. J Reprod Dev. 2015;61:145–153 25740096 10.1262/jrd.2014-081 PMC4410313

[84] Li HY, Liao CY, Lee KH, et al. Collagen IV significantly enhances migration and transplantation of embryonic stem cells: involvement of alpha2beta1 integrin-mediated actin remodeling. Cell Transplant. 2011;20:893–907 21176409 10.3727/096368910X550206

[85] Grzesiak JJ, Bouvet M The alpha2beta1 integrin mediates the malignant phenotype on type I collagen in pancreatic cancer cell lines. Br J Cancer. 2006;94:1311–1319 16622460 10.1038/sj.bjc.6603088 PMC2361410

[86] Diez-Torre A, Silvan U, De Wever O, et al. Germinal tumor invasion and the role of the testicular stroma. Int J Dev Biol. 2004;48:545–557 15349829 10.1387/ijdb.041897ad

[87] Yamana R, Iwasaki M, Wakabayashi M, et al. Rapid and deep profiling of human induced pluripotent stem cell proteome by one-shot NanoLC-MS/MS analysis with meter-scale monolithic silica columns. J Proteome Res. 2013;12:214–221 23210603 10.1021/pr300837u

[88] Kanematsu A, Marui A, Yamamoto S, et al. Type I collagen can function as a reservoir of basic fibroblast growth factor. J Control Release. 2004;99:281–292 15380637 10.1016/j.jconrel.2004.07.008

[89] Suh HN, Han HJ Collagen I regulates the self-renewal of mouse embryonic stem cells through alpha2beta1 integrin- and DDR1-dependent Bmi-1. J Cell Physiol. 2011;226:3422–3432 21344393 10.1002/jcp.22697

[90] Rodin S, Domogatskaya A, Strom S, et al. Long-term self-renewal of human pluripotent stem cells on human recombinant laminin-511. Nat Biotechnol. 2010;28:611–615 20512123 10.1038/nbt.1620

[91] Gu J, Fujibayashi A, Yamada KM, et al. Laminin-10/11 and fibronectin differentially prevent apoptosis induced by serum removal via phosphatidylinositol 3-kinase/Akt- and MEK1/ERK-dependent pathways. J Biol Chem. 2002;277:19922–19928 11891225 10.1074/jbc.M200383200

[92] Domogatskaya A, Rodin S, Boutaud A, et al. Laminin-511 but not -332, -111, or -411 enables mouse embryonic stem cell self-renewal in vitro. Stem Cells. 2008;26:2800–2809 18757303 10.1634/stemcells.2007-0389

[93] Semba K, Nishizawa M, Miyajima N, et al. yes-related protooncogene, syn, belongs to the protein-tyrosine kinase family. Proc Natl Acad Sci USA. 1986;83:5459–5463 3526330 10.1073/pnas.83.15.5459 PMC386306

[94] Baer AS, Syed YA, Kang SU, et al. Myelin-mediated inhibition of oligodendrocyte precursor differentiation can be overcome by pharmacological modulation of Fyn-RhoA and protein kinase C signalling. Brain. 2009;132:465–481 19208690 10.1093/brain/awn334 PMC2640211

[95] Liang X, Draghi NA, Resh MD Signaling from integrins to Fyn to Rho family GTPases regulates morphologic differentiation of oligodendrocytes. J Neurosci. 2004;24:7140–7149 15306647 10.1523/JNEUROSCI.5319-03.2004 PMC6729178

[96] Colognato H, Ramachandrappa S, Olsen IM, et al. Integrins direct Src family kinases to regulate distinct phases of oligodendrocyte development. J Cell Biol. 2004;167:365–375 15504915 10.1083/jcb.200404076 PMC2172535

[97] Sroka IC, Anderson TA, McDaniel KM, et al. The laminin binding integrin alpha6beta1 in prostate cancer perineural invasion. J Cell Physiol. 2010;224:283–288 20432448 10.1002/jcp.22149 PMC4816210

[98] Litjens SH, de Pereda JM, Sonnenberg A Current insights into the formation and breakdown of hemidesmosomes. Trends Cell Biol. 2006;16:376–383 16757171 10.1016/j.tcb.2006.05.004

[99] Klein S, Giancotti FG, Presta M, et al. Basic fibroblast growth factor modulates integrin expression in microvascular endothelial cells. Mol Biol Cell. 1993;4:973–982 8298194 10.1091/mbc.4.10.973 PMC275731

[100] Ruoslahti E, Pierschbacher MD New Perspectives in Cell-Adhesion - Rgd and Integrins. Science. 1987;238:491–497 2821619 10.1126/science.2821619

[101] Humphries JD, Byron A, Humphries MJ Integrin ligands at a glance. J Cell Sci. 2006;119:3901–3903 16988024 10.1242/jcs.03098 PMC3380273

[102] Ruoslahti E RGD and other recognition sequences for integrins. Ann Rev Cell Dev Biol. 1996;12:697–715 8970741 10.1146/annurev.cellbio.12.1.697

[103] Danen EH, Sonneveld P, Brakebusch C, et al. The fibronectin-binding integrins alpha5beta1 and alphavbeta3 differentially modulate RhoA-GTP loading, organization of cell matrix adhesions, and fibronectin fibrillogenesis. J Cell Biol. 2002;159:1071–1086 12486108 10.1083/jcb.200205014 PMC2173988

[104] Amit M, Shariki C, Margulets V, et al. Feeder layer- and serum-free culture of human embryonic stem cells. Biol Reprod. 2004;70:837–845 14627547 10.1095/biolreprod.103.021147

[105] Huveneers S, Truong H, Fassler R, et al. Binding of soluble fibronectin to integrin alpha5 beta1—link to focal adhesion redistribution and contractile shape. J Cell Sci. 2008;121:2452–2462 18611961 10.1242/jcs.033001

[106] Lee JW, Juliano RL The alpha5beta1 integrin selectively enhances epidermal growth factor signaling to the phosphatidylinositol-3-kinase/Akt pathway in intestinal epithelial cells. Biochim Biophys Acta. 2002;1542:23–31 11853876 10.1016/s0167-4889(01)00161-6

[107] Chang YQ, Li H, Guo ZK Mesenchymal Stem Cell-Like Properties in Fibroblasts. Cell Physiol Biochem. 2014;34:703–714 25171291 10.1159/000363035

[108] Farias E, Lu M, Li X, et al. Integrin alpha8beta1-fibronectin interactions promote cell survival via PI3 kinase pathway. Biochem Biophys Res Commun. 2005;329:305–311 15721307 10.1016/j.bbrc.2005.01.125

[109] Vogel BE, Tarone G, Giancotti FG, et al. A novel fibronectin receptor with an unexpected subunit composition (alpha v beta 1). J Biol Chem. 1990;265:5934–5937 2138612

[110] Bodary SC, Mclean JW The integrin beta-1 subunit associates with the vitronectin receptor alpha-V subunit to form a novel vitronectin receptor in a human embryonic kidney-cell line. J Biol Chem. 1990;265:5938–5941 1690718

[111] Aplin JD, Sattar A, Mould AP Variant choriocarcinoma (BeWo) cells that differ in adhesion and migration on fibronectin display conserved patterns of integrin expression. J Cell Sci. 1992;103:435–444 1478945 10.1242/jcs.103.2.435

[112] Dedhar S, Gray V Isolation of a novel integrin receptor mediating Arg-Gly-Asp directed cell-adhesion to fibronectin and type-I collagen from human neuroblastoma-cells—association of a novel beta-1-related subunit with alpha-V. J Cell Biol. 1990;110:2185–2193 1693626 10.1083/jcb.110.6.2185 PMC2116123

[113] Marshall JF, Rutherford DC, Mccartney ACE, et al. Alpha(V)Beta(1) is a receptor for vitronectin and fibrinogen, and acts with alpha(5)beta(1) to mediate spreading on fibronectin. J Cell Sci. 1995;108:1227–1238 7542669 10.1242/jcs.108.3.1227

[114] Munger JS, Huang X, Kawakatsu H, et al. The integrin alpha v beta 6 binds and activates latent TGF beta 1: a mechanism for regulating pulmonary inflammation and fibrosis. Cell. 1999;96:319–328 10025398 10.1016/s0092-8674(00)80545-0

[115] Prowse AB, Doran MR, Cooper-White JJ, et al. Long term culture of human embryonic stem cells on recombinant vitronectin in ascorbate free media. Biomaterials. 2010;31:8281–8288 20674971 10.1016/j.biomaterials.2010.07.037

[116] Aluwihare P, Mu ZY, Zhao ZC, et al. Mice that lack activity of alpha v beta 6-and alpha v beta 8-integrins reproduce the abnormalities of Tgfb1- and Tgfb3-null mice. J Cell Sci. 2009;122:227–232 19118215 10.1242/jcs.035246 PMC2714418

[117] Yang ZW, Mu ZY, Dabovic B, et al. Absence of integrin-mediated TGF beta 1 activation in vivo recapitulates the phenotype of TGF beta 1-null mice. J Cell Biol. 2007;176:787–793 17353357 10.1083/jcb.200611044 PMC2064053

